# Sprint interval climbing increases anaerobic training load in elite female +78 kg judo athletes

**DOI:** 10.3389/fspor.2025.1581361

**Published:** 2025-07-17

**Authors:** Liying Huang, Hu Yao, Xiquan Weng, Hao Chen

**Affiliations:** ^1^ College of Exercise and Health, Guangzhou Sport University, Guangzhou, China; ^2^ Guangdong Provincial Key Laboratory of Human Sports Performance Science, Guangzhou Sport University, Guangzhou, China; ^3^ College of Sports Science, Zhuhai College of Science and Technology, Zhuhai, Guangdong Province, China; ^4^ Scientific Research Center, Guangzhou Sport University, Guangzhou, China

**Keywords:** judo training, heavy weight, HIIT, anaerobic, versaclimber

## Abstract

**Introduction:**

The decisive movements that determine judo performance rely on anaerobic power. Currently, the optimum training protocol for increasing the anaerobic endurance of heavyweight judo athletes remains elusive due to tricky balance between training loads increase and injury prevention. This study investigated the impact of sprint interval climbing incorporated into the regular training on the anaerobic training load of heavyweight judo athletes.

**Methods:**

Five judo athletes of the female +78 kg category from the Chinese national team (average age: 26.8 ± 2.8 years; height: 185.6 ± 5.7 cm; weight: 127.8 ± 5.8 kg; judo training experience: 15 ± 3.5 years) conducted only regular training from October to December 2019 while climbing training was added to regular training from January to March 2020. A climbing fitness test was performed once per month from January to March 2020. The anaerobic training loads in the training and simulated matches were monitored through blood lactate and heart rate metrics, the physical fitness of the subjects was monitored through blood metrics such as creatine kinase, and the internal load of the subjects was assessed using Omegawave Technology readiness scores.

**Results:**

We found that sprint interval climbing increased the levels of blood lactate (*P* = 0.00) and heart rate metrics (*P* = 0.00) in the training and/or simulated matches and the performance in the fitness tests. Meanwhile, physiological parameters and the internal load remained comparable before and after climbing training.

**Conclusions:**

These results suggested that sprint interval climbing increased anaerobic training load without obvious muscular damage or fitness decrease. Finally, lack of a control group due to limited availability of the subjects meeting the criteria and the need to maximize the performance of each subject in future matches was the major limitation of this study.

## Introduction

Although judo relies primarily on aerobic power, the decisive movements that determine its performance depend on the anaerobic power (1, 2). In addition, although the upper limbs' muscles are usually the most involved for certain techniques, e.g., kumi-kata in a judo combat, explosive and decisive actions are performed by the lower limbs (2, 3). Therefore, developing anaerobic endurance of both upper and lower limbs is central to judo training (4, 5). However, a widely accepted program for judo anaerobic endurance training seems to be lacking so far (2, 6). Besides, as judo generally serves as high-intensity interval exercise (2, 4), high-intensity interval training (HIIT) through running (7, 8) or ergometer cycling (4, 5) is widely applied in judo training.

Our team has been long involved in monitoring the training of the athletes of the female +78 kg category from the Chinese national judo team. These athletes generally weigh over 100 kg, which prevents them substantially from heavy load training. For instance, due to frequent occurrence of knee injury during high-intensity interval running (9, 10), the training load of these athletes have to be increased cautiously, which may affect the training effect. Although high-intensity interval ergometer cycling can serve as an alternative to reduce the pressure on athlete knees and thus probable knee injuries, it cannot simultaneously increase the anaerobic endurance training load on both upper and lower limbs (4, 5). Therefore, the optimum training protocol for increasing the anaerobic endurance of the judo athletes of the +78 kg class remains to be further explored.

Indoor simulated climbing using e.g., Versaclimber is constituted by impact free, smooth motions which can decrease the likeliness of injury to joints such as knees (11, 12). In addition, the arms and legs are being utilized in an alternating push-pull motion during Versaclimber training (13–15). This style not only trains both upper and lower limbs simultaneously, but also meets the technical requirement of the judo combat as push-pull motions frequently occur in this combat sport (1, 2). Moreover, interval training can also be achieved on Versaclimber by sprinting (13, 15). Considering the advantages mentioned above, interval Versaclimber sprinting was incorporated into the regular training of the female +78 kg athletes of the Chinese national judo team during their training before the Tokyo Olympics, and the impact of such a training on the anaerobic and aerobic training loads was investigated.

## Methods

### Study design

The subjects of this study were five female +78 kg judo athletes of the Chinese national judo team. As this study was conducted during the training for the 2020 Tokyo Olympics, the training program needed to follow the schedule of the national team. In this study lasting half a year, the subjects conducted regular training from October to December 2019 while Versaclimber training was added to regular training from January to March 2020. The daily training time remained the same in this study. The subjects performed 2–3 simulated matches per week. In addition, they conducted a climbing fitness test once per month from January to March 2020. A regular diet and schedule were maintained for the subjects to prevent injuries and illnesses during this study. The anaerobic training load was monitored predominantly through blood lactate and heart rate metrics such as training impulse (TRIMP), the physical fitness of the subjects was monitored through metrics such as creatine kinase (CK) and blood testosterone (T), and the internal pressure of the subjects was assessed using Omegawave Technology readiness scores [(16), Omegawave Ltd., Espoo, Finland].

### Subjects

This study recruited five top-level judo athletes of the female +78 kg category from the Chinese national judo team (average age: 26.8 ± 2.8 years; height: 185.6 ± 5.7 cm; weight: 127.8 ± 5.8 kg; judo training experience: 15 ± 3.5 years). The inclusion criteria were as follows: (a) the athlete had to be a member of the Chinese national judo team and performed training following the instructions of the team throughout the study; (b) the athlete had to be free of injury and healthy and complete all planned training sessions; (c) the athlete did not undergo climbing training before. Written consent was obtained from all participants after they were informed about the experimental design. This study has been approved by the Guangzhou Sport University Ethics Committee. All procedures were conducted in accordance with the Helsinki Declaration on Human studies.

### Versaclimber training program

Three sprinting sessions on Versaclimber (CL-108SM, Santa Ana, CA) were added to the regular training on nonconsecutive days per week from January to March 2020, and the training volume/intensity remained the same during this period. Each session consisted of two 1-minute sprints, two 2-minute sprints and one 4-minute sprint with a 4-minute interval between each sprint. After sufficient warm-up, the subjects underwent climbing on Versaclimber with the “faster step” resistance mode. Earlobe blood was collected immediately, 1, 3, 5 min after the climbing and subjected to examination to determine the lactate level on a portable blood lactate meter (EKF Diagnostics, United Kingdom). Blood CK was examined after daily training through the DXC600 chemistry analyzer [Beckman Coulter Commercial Enterprise (China) Co., Ltd, China]. Blood urea was examined after daily training through Reflotron Plus Dry dry chemistry analyzer (Roche Diagnostics, Switzerland). Serum testosterone and cortisol (C) were examined before breakfast through DXI800 immunoassay analyzer [Beckman Coulter Commercial Enterprise (China) Co., Ltd., China]. Omegawave Technology readiness scores were examined when the subjects were in sedentary states in the morning once per month for each subject.

### Simulated matches

Each match lasted 4 min. The combats were mediated according to official rules, and the opponents were selected randomly among the subjects. Earlobe blood was collected immediately, 1, 3, and 5 min after the combats to determine the lactate level on a portable EKF blood lactate meter as stated above. The anaerobic training load was also monitored through FirstBeat SPORT heart rate monitor (FirstBeat SPORT, Finland). Key metrics observed through FirstBeat included maximum heart rate percentage (%HRmax), heart rate zone, training effectiveness (TE), excess post-exercise oxygen consumption (EPOC) and TRIMP.

### Climbing fitness test

After sufficient warm-up, the subjects wore a FirstBeat SPORT heart rate monitor and conducted a 2-minute sprint on the Versaclimber with the “faster step” resistance mode. Fitness was evaluated through the following metrics: climbing distance (m), average heart rate (average HR, beats/minute), maximum heart rate (HRmax, beats/minute), the time above 80% of the HRmax (seconds) and Fitness Score. Fitness Score = the time above 80% of the HRmax × 100/113. 113 (seconds) was from 120 s minus 7 s in which 120 s (2 min) was the time for the test while 7 s was the time for the subject's HR to get to 80% of her HRmax from the beginning of the test on the Versaclimber generally. Therefore, Fitness Score was equivalent to the time above 80% of the HRmax. This formula was provided by the General Administration of Sport of China. Scores over 100 were deemed to be 100.

## Statistics

The data in this study were analyzed using Graphpad prism 9.0 and SPSS26.0, and the values were expressed as mean ± standard deviation (M ± SD). Data were tested for normality and homogeneity of variance using a Shapiro–Wilk and Levene's test, respectively. Student's *t*-test was used for pairwise comparison while one-way analyses of variance (ANOVAs) with Fisher's LSD *post hoc* tests were used to analyze and compare the climbing fitness test data. Partial eta-squared (*η*^2^*_p_*) was used to estimate effect size, and values were interpreted as no effect (*η*^2^*_p_* < 0.04), minimum effect (0.04 < *η*^2^*_p_* < 0.25), moderate effect (0.25 < *η*^2^*_p_* < 0.64), or strong effect (*η*^2^*_p_* > 0.64) (6). ANOVAs with Sidak or Tukey's *post hoc* tests were used to analyze and compare blood lactate concentrations after climbing training and simulated matches conducted from January to March 2020. *P*-values less than 0.05 were considered statistically significant.

## Results

### Anaerobic training load

**Figure 1:**
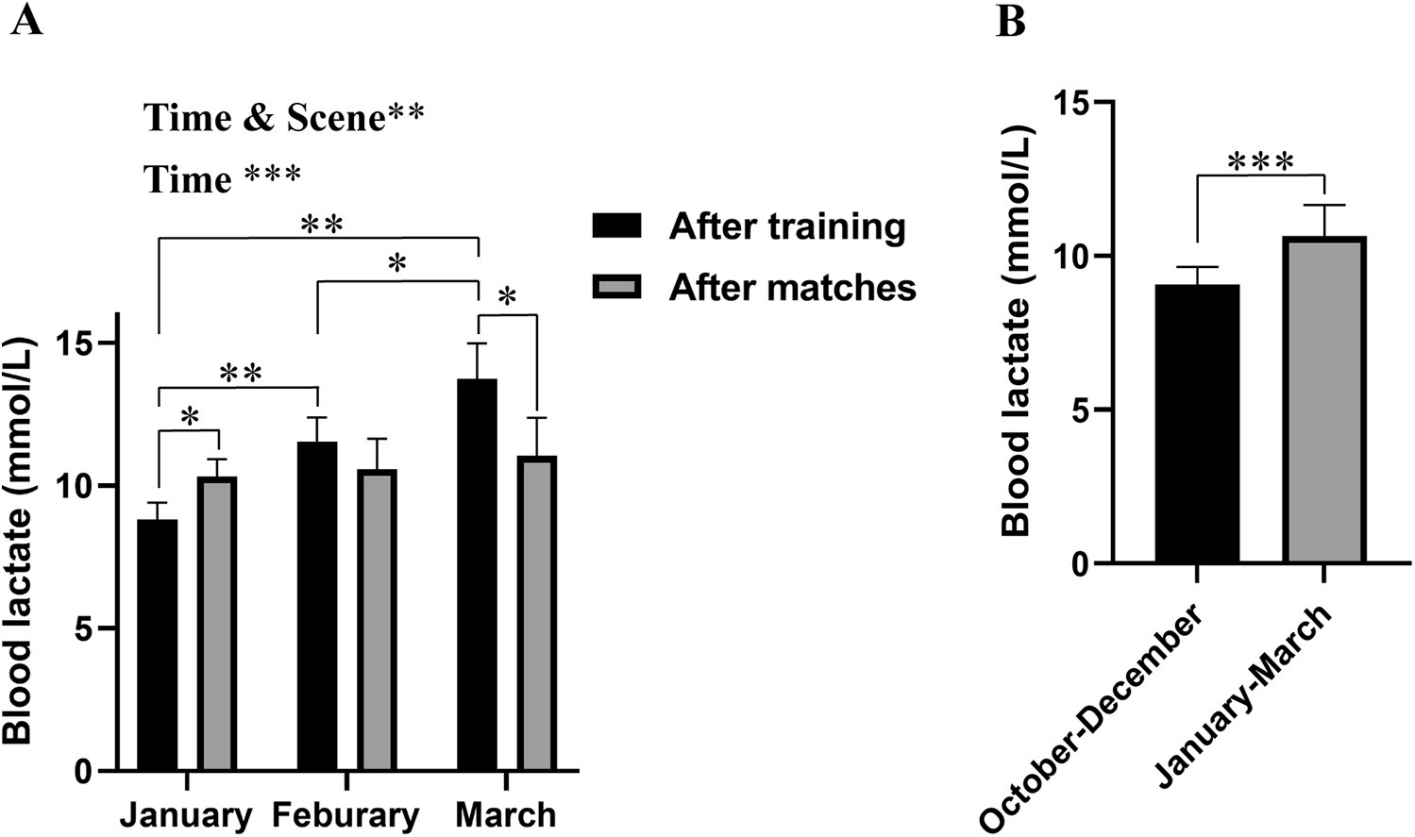
Blood lactate concentrations during the study. **(A)** Blood lactate concentrations were examined after climbing training or simulated matches conducted from January to March 2020 and two-way repeated measures ANOVAs with Sidak or Tukey *post hoc* tests were used to compare the data; **(B)** Blood lactate concentrations were examined after simulated matches conducted from October to December 2019 or from January to March 2020 and student's *t*-test was used for pairwise comparison. Data were expressed as mean ± SD. *, **, and *** denote a significant difference for interaction, main effect or between groups of *P* < 0.05, *P* < 0.01 and *P* < 0.001, respectively.

As shown in Figure 1A, the levels of blood lactate upon climbing training were increased gradually from January to March 2020 (Mean difference for January vs. February: −2.740, 95% CI of difference = −4.344 to −1.136, *p* = 0.0081; Mean difference for February vs. March: −2.180, 95% CI of difference = −4.172 to −0.1880, *p* = 0.0376). In addition, the levels of blood lactate upon simulated matches displayed a similar trend from January to March 2020 although the increase was statistically insignificant (Figure 1A). Accordingly, while the level of blood lactate upon climbing training was lower than that upon simulated match in January (Mean difference for “after training” vs. “after matches” = −1.500, 95% CI of difference = −2.639 to −0.3605, *p* = 0.0126) or comparable to that in Feburay 2020, respectively, it became higher than that in March 2020 (Mean difference for “after training” vs. “after matches” = 2.700, 95% CI of difference = 0.2414–5.159, *p* = 0.0323; Figure 1A). Likewise, the level of blood lactate upon the three-month climbing training was higher than that upon the previous three-month period without climbing training (*p* = 0.0002, *η*^2^*_p_* = 0.6475, strong effect; Figure 1B).

In addition to the blood lactate level, the training load of the athletes in the simulated matches was also monitored through the FirstBeat SPORT heart rate monitor system. As shown in Table 1, not only two core metrics TRIMP and EPOC but also three %HRmax-related parameters were all increased after the three-month climbing training. As the anaerobic energy system serves as the predominant source for energy when %HRmax is over 80% (17), the increased percentages of time spent in both 80%–90% and 90%–100% HRmax suggested that the anaerobic training load in the athletes might be increased in the simulated matches after the three-month climbing training.

**Table 1:** Parameters in the simulated matches monitored through FirstBeat SPORT heart rate monitor.

FirstBeat parameters	From October to December	From January to March	*P*-value
%HRmax	87 ± 4.8	91 ± 3.8	0.00
Percentage of time spent in 80%–90% HRmax	11.8 ± 8.7	17.8 ± 10.0	0.00
Percentage of time spent in 90%–100% HRmax	0.8 ± 1.9	3.5 ± 4.4	0.00
TRIMP	97.6 ± 41	117.7 ± 26.4	0.00
EPOC	41.9 ± 20	73.0 ± 23.5	0.00

Data were expressed as mean ± SD. *P* < 0.05 indicated that the difference was statistically significant.

### Versaclimber-based fitness test

**Figure 2:**
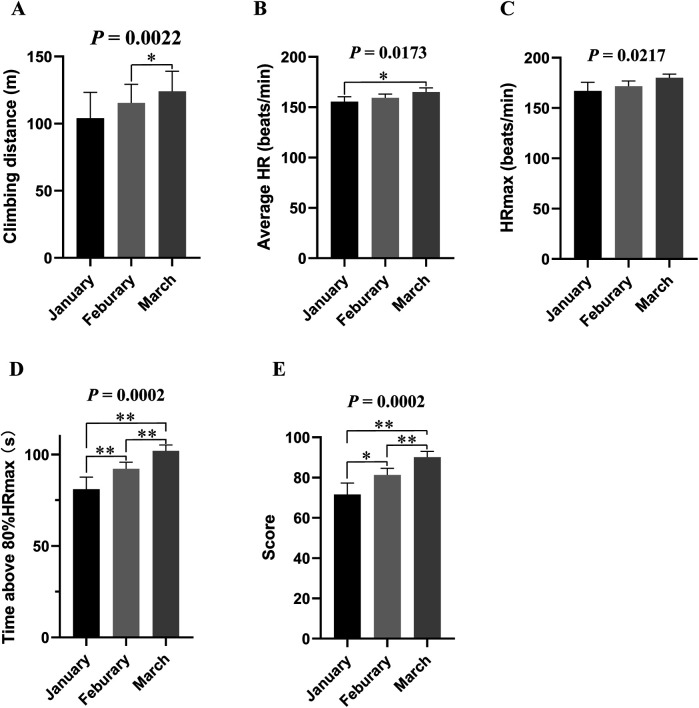
The parameters of the climbing fitness test. **(A)** Climbing distance; **(B)** Average heart rate; **(C)** Maximum heart rate (HRmax, beats/minute); **(D)** Time with at least 80% of the maximum heart rate; **(E)** Fitness Score. One-way ANOVAs with Fisher's LSD *post hoc* tests were used to analyze and compare the climbing fitness test data. Data were expressed as mean ± SD. *, **, and *** denote a significant difference between groups of *P* < 0.05, *P* < 0.01 and *P* < 0.001, respectively.

As shown in Figure 2, while all the metrics were gradually increased from January to March 2020, the increase of the two metrics including time above 80%HRmax (*p* = 0.0002, *η*^2^*_p_* = 0.9405, strong effect; Figure 2D) and test score (*p* = 0.0002, *η*^2^*_p_* = 0.9349, strong effect; Figure 2E) was the most significant. Notably, the increases of average HR and HRmax suggested that the aerobic training load was also enhanced.

### Physiological response and internal training load

To avoid overtraining after adding the climbing training to the regular training, several blood parameters other than lactate were also monitored throughout the study. As shown in Table 2, no significant differences were observed for CK, BU, T, C and T/C upon climbing training. In addition, the internal training load of the athlete during this period was also monitored through the Omegawave technology. As shown in Table 3, majority of the metrics did not change significantly upon climbing training except Metabolic Reaction Index and Anaerobic Readiness.

**Table 2:** Blood parameters.

Blood parameters	From October to December	From January to March	*P*-value
Creatine kinase (CK, U/L)	478.27 ± 108.17	518.07 ± 164.47	0.29
Blood urea (mmol/L)	7.65 ± 1.61	7.96 ± 2.15	0.49
Testosterone (T, ng/dl)	57.81 ± 6.95	61.14 ± 8.9	0.29
Cortisol (C, ug/dl)	14.93 ± 2.23	16.02 ± 2.00	0.19
T/C	3.95 ± 0.73	3.86 ± 0.64	0.72

Data were expressed as mean ± SD. *P* < 0.05 indicated that the difference was statistically significant.

**Table 3:** Omegawave technology fitness scores.

Omegawave parameters	From October to December	From January to March	*P*-value
Metabolic reaction index	187.14 ± 44.06	200.06 ± 22.51	0.04
Anaerobic readiness	137.95 ± 2.83	139.01 ± 2.36	0.04
Omegawave readiness	6.00 ± 1.00	6.22 ± 0.80	0.12
Cardiac system readiness	6.31 ± 0.93	6.5 ± 0.72	0.30
Central nervous system	6.00 ± 0.84	5.98 ± 1.14	0.92

Data were expressed as mean ± SD. *P* < 0.05 indicated that the difference was statistically significant.

## Discussion

Judo athletes of different weight categories display divergent anthropometric and physiological parameters (1, 18), which warrants specific training programs for different weight categories (19, 20). However, previous studies pay more attention to adaptation to weight control (21, 22) while few explore adaptation to training programs for different weight categories so far. Recently, Kashiwagura et al. found that judo athletes of different weight categories display different preferences in gripping during attacks (20), which can help coaches to develop the best training strategies specific for different weight categories. However, few studies explored the strategies specific for various weight categories to enhance anaerobic or aerobic capacities in judo training. Particularly, as strength and body mass tend to be inversely related (1, 19), it's more mandatory to develop an effective strategy to further enhance the anaerobic capacity of the heaviest judo athletes. Meanwhile, heavyweight judo athletes are prone to suffer from more injuries, particularly knee injuries, than lightweight judo athletes (9, 10). As training load is one of the key aspect related to athlete injury (23, 24), it is mandatory to coordinate the training load of the heavyweight judo athletes to avoid injuries while increasing their performance. However, studies in which the training loads of heavyweight judo athletes were monitored were largely lacking so far. In this study, the load increase in the judo athletes of the female +78 weight category was verified or monitored through blood lactate or heart-rate-based metrics. Meanwhile, physiological parameters including T/C ratio, CK and BU remained comparable before and after climbing training. These results indicated that although the anaerobic training load imposed on the subjects during climbing training matched or even exceeded that during simulated matches, obvious muscular damage or fitness decrease seemed not to occur. This result suggested that the athletes had positive adaptation to the training load. Our observation was consistent with the study of Marques et al. in which the judo athletes display increases in judo-specific performance without significant changes in cortisol and testosterone concentrations during block periodization training (25).

In this report, we used Omegawave technology to monitor internal training load in the athlete before and during the introduction of climbing training (16). The three key metrics of the Omegawave technology including Omegawave Readiness, Cardiac System Readiness and Central Nervous System (Table 3) did not change significantly upon climbing training. This result suggested that the internal training loads remained generally unchanged and further supported that the athletes had positive adaptation to the training loads. This result was consistent with a previous study in which the judo athletes displayed no difference in the internal training load over 4 weeks of HIIT added to regular judo training although the internal training load was investigated through subjective rating of perceived exertion (RPE) in this study (5). Besides, two metrics of the Omegawave technology metabolic reaction index and anaerobic readiness were also increased upon climbing training, further supporting the increased anaerobic training load.

This study aimed at increasing the anaerobic training load in the judo athletes of the female +78 weight category, which was validated by the increased level of blood lactate, the direct marker of the anaerobic glycolytic system, during and upon the climbing training. This observation was consistent with previous studies (5, 26). However, notably, the level of blood lactate is not increased in certain other studies in which anaerobic exercise is also introduced into the regular training of judo athletes (4, 6). These different results may be attributed to different levels of stress onanaerobic metabolism conferred by different training protocols (2), different durations of training periods (26), or different movement efficiency achieved upon different training protocols (6) in different subjects (2, 6). In addition, interestingly, although the levels of blood lactate upon simulated matches tended to increase in the three-month climbing training, the increase was statistically insignificant and much less than that of the levels of blood lactate upon climbing training. This difference suggested a dilemma that although the anaerobic power of the subjects might be enhanced as revealed by the increased blood lactate upon the climbing training, this enhancement was not displayed significantly in the blood lactate level upon the simulated matches. This may be concerned with the difference between the climbing training protocol and the time-motion situation in the simulated matches. In this study, longer time (≥1 min) for each set was used in the training as did in certain HIIT judo training studies (27–30) while simulated matches were actually constituted by combat-pause cycles of shorter time (around 20 s per combat with an interval around 10 s) (2, 31). Therefore, it was possible that the overall workload of the anaerobic glycolytic system in the simulated matches might not be as high as that in the climbing training, which may help to explain the less increased lactate level upon the simulated matches. On the other hand, notably, the lactate levels in certain judo HIIT training studies (4, 32) in which less time were used for each set (20–30 s for a training set with a 5–10-s interruption) were not increased significantly, suggesting that the overall training load may not be high enough. Therefore, an equilibrium between increasing the blood lactate level and meeting the time-motion of the judo match in training protocol still awaited further exploration in future studies.

Like many other HIIT studies (4, 8), the HIIT protocol in this study aimed to increase anaerobic load might also increase aerobic load in the athletes as revealed by the fitness test, although the increase was not as significant as that of the anaerobic load. However, notably, certain interval training studies in judo displayed only marked increases in anaerobic parameters in the athletes without increases in aerobic ones (6, 7). Like the differences on the lactate level mentioned above, this difference may be attributed to different levels of stress on aerobic metabolism conferred by different training protocols or different durations of the same protocol in different subjects. For instance, Franchini et al., found that the subjects display an increase in upper-body maximal aerobic power upon 4-week upper-body HIIT training on the cycle-ergometer while Kim et al., demonstrate that elite judoists display no significant changes in aerobic performance upon 4-week or even 8-week sprint interval running on a treadmill (7). However, Kim's protocol seems to lead to improvement in aerobic performance after 12 weeks' training in the same subjects (8). The precise mechanism for different outcomes on aerobic performance remains unknown. Clarification of this issue may necessitate quantification of the stress on and of the adaptation of the aerobic energy system.

This study demonstrated the introduction of a protocol of sprint interval training on Versaclimber to the regular training of five elite female +78 kg judo athletes, resulting in significant improvements in the anaerobic training load without obvious muscular damage or fitness decrease. This protocol can be potentially applied to the training of anaerobic power of heavyweight judo athletes who need to manage their anaerobic training load and prevent from probable joint injuries.

Finally, two limitations of this study had to be acknowledged. First of all, lack of a control group was the major limitation of this study. As only the top-level female +78 kg judo athletes in the national team were subjected to comprehensive training mornitoring, we were not able to recruit more than five subjects, nor a control group. However, because the subjects had similar competition levels and training histories, we observed similar physiological parameters among them and similar response to the sprint climbing training. Besides, since this study was conducted during the training period before the Tokyo 2020 Summer Olympics, the subjects needed a uniform intervention for a fair competition, which made us unable to set a control group. Regardless, future studies with a bigger sample size and a control group are warranted. Secondly, as the climbing training was conducted during the intervals of regular training, the resulting effects of the climbing training may result cumulatively from the climbing and regular training, although numerous training load markers included in this report showed uniformed increases after the climbing training was introduced into the regular training. Whether similar results could be observed with the exclusive application of climbing training over a three-month period remains to be further elucidated. It cannot be excluded that more frequent or intensive training may be warrant to observe the effect described in this report.

## Data Availability

The raw data supporting the conclusions of this article will be made available by the authors, without undue reservation.
